# October issue of 2012, vol. 99(4), pages 1-10

**DOI:** 10.5935/abc.20190091

**Published:** 2019-05

In "Brazilian Guidelines for Vehicular Direction in Implantable Cardiac Devices and Cardiac Arrhythmias" (portuguese: Diretrizes Brasileiras para Direção Veicular em Portadores de Dispositivos Cardíacos Eletrônicos Implantáveis e Arritmias Cardíacas), page 2, second paragraph, consider correct for the phrase "suspension of driving for six months" (portuguese: suspensão da direção por seis meses) three months in place of six months.

